# Synthesis of Polypyrrole Induced by [Fe(CN)_6_]^3−^ and Redox Cycling of [Fe(CN)_6_]^4−^/[Fe(CN)_6_]^3−^

**DOI:** 10.3390/polym10070749

**Published:** 2018-07-06

**Authors:** Eivydas Andriukonis, Almira Ramanaviciene, Arunas Ramanavicius

**Affiliations:** 1 Department of Physical Chemistry, Faculty of Chemistry and Geosciences, Vilnius University, Naugarduko 24, LT-03225 Vilnius, Lithuania; eivydas.andriukonis@chgf.vu.lt; 2 NanoTechnas, Center of Nanotechnology and Materials Science, Faculty of Chemistry and Geosciences, Vilnius University, Naugarduko 24, LT-03225 Vilnius, Lithuania; almira.ramanaviciene@chf.vu.lt; 3 Laboratory of Nanotechnology, Center for Physical Sciences and Technology, State Research Institute, Sauletekio Ave. 3, LT-10257 Vilnius, Lithuania

**Keywords:** conducting polymers, polypyrrole, polymerization reaction, reaction kinetics, redox cycling, initiator of polymerization

## Abstract

Chemical synthesis of the conducting polymer polypyrrole induced by [Fe(CN)_6_]^3−^ is reported. Reaction kinetics were characterized spectrophotometrically. Reaction rate was evaluated at several different pH levels in the presence of [Fe(CN)_6_]^3−^ and [Fe(CN)_6_]^4−^ ions. The formation of polypyrrole at aerobic and anaerobic conditions was evaluated. We report that at anaerobic conditions [Fe(CN)_6_]^4−^ cannot initiate oxidative polymerization, while its oxidized form [Fe(CN)_6_]^3−^ successfully initiates and maintains the pyrrole polymerization reaction. The formation of polypyrrole was also observed in the solution containing a pyrrole monomer, [Fe(CN)_6_]^4−^ and dissolved oxygen due to re-oxidation (redox cycling) of [Fe(CN)_6_]^4−^ into [Fe(CN)_6_]^3−^ by dissolved oxygen. Experiments to determine the polymerization reaction rate were performed and showed the highest rate in the presence of 0.5 mM of [Fe(CN)_6_]^3−^ at pH 9.0, while the polymerization reaction performed at pH 7.0 was determined as the slowest. This investigation opens new horizons for the application of [Fe(CN)_6_]^4−^/[Fe(CN)_6_]^3−^-based redox cycling reactions in the synthesis of the conducting polymer polypyrrole and potentially in the formation of other conducting polymers which can be formed by oxidative polymerization.

## 1. Introduction

Conducting polymers have been a subject of great interest over the last 20 years. They are applied as conductors or semiconductors in many technological areas including the design of sensors and biosensors [1,2]. Among conducting polymers polypyrrole (Ppy) is one of the most widely investigated due to its excellent environmental and thermal stability, good electrical conductivity, useful optical properties, and insolubility in common organic solvents [3]. Polypyrrole has found attractive applications as one of the fundamental building materials in the design of various electronic analytical tools, solar cells, and light-weight batteries. It has attracted much interest in the development of biosensors based on immobilized biological substances in various fields such as health care, immunosensors, DNA sensors, biosensors, environmental monitoring, and food analysis [3,4]. In biosensors polypyrrole is used as an effective matrix for the immobilization of redox compounds [5,6], enzymes [7,8,9,10], DNA [11,12] or antibodies [11].

Synthesis of polypyrrole can be performed in many ways. The most efficient methods of conducting polymer synthesis are based on (i) chemical oxidative polymerization [6,12,13,14,15,16], (ii) electrochemical polymerization [17], (iii) enzymatic polymerization [7,8,9,10], and (iv) UV-induced polymerization [18]. Significant differences between Ppy formed by the above mentioned synthesis methods allow the formation of a wide variety of different Ppy forms ranging from simple semi-conducting polymer films to polymeric nanoparticles [19,20]. It should be noted that using the listed methods not only polypyrrole but similar conducting polymers such as polyaniline [21] and polythiophene [22] can also be synthesized. However, among these conducting polymers Ppy is the most interesting due to its high biocompatibility and possible application in biomedicine [12,20,23].

Various oxidants including FeCl_3_, (NH_4_)_2_S_2_O_8_ and H_2_O_2_ are used as initiators of the chemical synthesis of polypyrrole [9,24,25,26]. These oxidants are mostly used for the chemical formation of Ppy. In the presence of the above mentioned oxidisers the formation of Ppy is very fast due to the relatively high oxidation potential of the oxidants. The polymerization reaction rate is very important for the control of particle size, shape, and some other properties [27]. In some particular cases the polymerization rate is controlled by the variation of temperature, but other parameters as size and shape are rarely controlled this way [28]. From a technological point of view it is important to determine relatively mild oxidative conditions suitable for the chemical synthesis of Ppy which will allow slower and better controlled formation of Ppy. K_3_[Fe(CN)_6_] was chosen as an oxidizer because it has smaller oxidation potential in water (~+0.4 V) compared to that of FeCl_3_ or H_2_O_2_ (approximately +0.7 V).

All chemical and physical properties of K_3_[Fe(CN)_6_]/K_4_[Fe(CN)_6_] are well known. Both oxidized and reduced forms of this redox-pair are easily traceable with optical and electrochemical techniques. This complex salt is able to act as a redox couple [Fe(CN)_6_]^3−^/[Fe(CN)_6_]^4−^ and is very often used as a soluble redox mediator in the design of amperometric biosensors [29] and biofuel cells [30,31,32,33]. In several studies this redox compound was applied as a dopant of a Ppy layer [34,35,36]. In most cases either other oxidizing agents were used as pyrrole polymerization initiators or an external potential had been applied to initiate the formation of Ppy. Up until now little research was dedicated to the application of K_3_[Fe(CN)_6_] in Ppy synthesis [6,37,38]. Therefore, not all aspects of K_3_[Fe(CN)_6_]-initiated Ppy synthesis were evaluated. Altogether Ppy synthesis induced by K_3_[Fe(CN)_6_] can be very promising and technologically useful due to direct synthesis and doping of the formed Ppy with [Fe(CN)_6_]^3−^ or [Fe(CN)_6_]^4−^ ions.

In this research we investigated the ability of [Fe(CN)_6_]^3−^ to initiate the synthesis of Ppy. The possibility of exploiting the redox cycling of [Fe(CN)_6_]^3−^/[Fe(CN)_6_]^4−^ by dissolved oxygen during the synthesis of Ppy has been evaluated. The influence of pH on polymerization reaction kinetics has been investigated.

## 2. Methodology

### 2.1. Reagents and Materials

Potassium hexacyanoferrate (II) trihydrate (K_4_[Fe(CN)_6_]·3H_2_O, in further text depicted as [Fe(CN)_6_]^4−^, was purchased from Carl Roth GmbH&Co (Karlsruhe, Germany). Pyrrole (Py) (98%) was purchased from Sigma-Aldrich (St. Luis, MO, USA) and Fluka Chemie GmbH (Buchs, Switzerland). Sodium phosphate dibasic and potassium hexacyanoferrate(III) K_4_[Fe(CN)_6_], depicted as [Fe(CN)_6_]^3−^, were purchased from Sigma-Aldrich Laborchemikalien GmbH (Seelze, Germany).

### 2.2. Apparatus 

The spectrophotometric measurements were performed with a Lambda 25 UV–VIS spectrophotometer and a Spectrum 100 Fourier transform infrared spectroscope (FTIR) from Perkin Elmer (Waltham, MA, USA) using UV-cuvettes from semi-micro (Brand^®^) (Wertheim, Germany). Particles and aggregates were inspected with a Helios nanolap 650 focused ion beam scanning electron microscope (FIB SEM, Hillsboro, OR USA) and a Bruker Autoflex Max matrix-assisted laser desorption/ionization time of flight spectroscope (MALDI ToF, Karlsruhe, Germany).

### 2.3. Evaluation of Ppy Synthesis Initiated by [Fe(CN)_6_]^3−^

The standard solutions used in this research consisted of 0.1 M phosphate buffered saline (PBS) of different pHs with 0.04–0.1 M [Fe(CN)_6_]^3−^ and/or [Fe(CN)_6_]^4−^. Visual test of Ppy formation was performed at 0.04 M concentration of [Fe(CN)_6_]^3−^ or [Fe(CN)_6_]^4−^ with 0.5 M of pyrrole. Exact compositions of the solutions and visual evaluation of polymerization are indicated in Table 1. The same conditions were used to prepare Ppy for experiments with FTIR, matrix-assisted laser desorption/ionization time of flight (MALDI ToF), and focused ion beam scanning electron microscope (FIB SEM). The reaction was performed for 24 h with the reaction mixture mixed by an orbital shaker at 200 revolutions per minute at 30 °C. The synthesized polymer was centrifuged and supernatant (reaction mixture) was decanted. Collected sediments were washed multiple times using 1 mL of distilled water. Afterwards, the polymer was dried by being heated to 90 °C under a gentle stream of nitrogen gas. Dried samples were then analysed with an infrared spectrometer.

**Figure 1:**
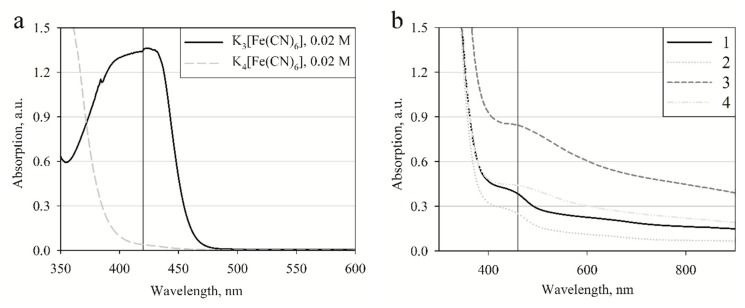
(**a**) Optical absorbance spectra of 0.02 M of [Fe(CN)_6_]^3−^ and 0.02 M of [Fe(CN)_6_]^4−^. The vertical line indicates the absorption maximum at 420 nm; (**b**) Optical absorbance spectra of Ppy synthesized under different conditions: (1) polymerization solution containing 0.5 M of pyrrole and 0.04 M of [Fe(CN)_6_]^3−^, spectrum was registered after 24 h from the initialization of the polymerization reaction, (2) supernatant collected after 24 h of polymerization followed by centrifugation at 12,000 *g* for 10 min, (3) 0.375 M of pyrrole and 0.1 M of [Fe(CN)_6_]^3−^ spectrum registered after 24 h of polymerization, (4) 1.5 M of pyrrole and 0.25 M of [Fe(CN)_6_]^3−^ spectrum registered after 24 h of polymerization. All spectra were registered in a 0.1 M PBS, pH 7.0. The vertical line indicates poorly expressed absorption maximum at 460 nm.

The samples were prepared by mixing pre-calculated amounts of initial standard solutions directly before experiments. Reactions were performed in different pHs PBS solutions usually containing 0.5 mM [Fe(CN)_6_]^3−^ or [Fe(CN)_6_]^4−^ and a 0.5 M concentration of Py. The polymerization was studied at three different [Fe(CN)_6_]^4−^ concentrations of 0.1, 0.5 and 1 mM and at two [Fe(CN)_6_]^3−^ concentrations of 0.1 and 0.5 mM, at pH 3.0, 5.0, 7.0 and 9.0. The kinetics of the reactions were evaluated by measuring the absorption at 420 nm and 460 nm wavelengths, which are related to optical absorption of [Fe(CN)_6_]^3−^ [39] (Figure 1a) and Ppy [9] (Figure 1b). The molar coefficient of optical extinction for [Fe(CN)_6_]^3−^ is 1179 mol^−1^∙dm^3^ cm^−1^, determined experimentally, while other authors describe the slightly lower value of 1040 mol^−1^∙dm^3^ cm^−1^ [40]. [Fe(CN)_6_]^4−^ did not show any significant absorption maximum in the visible light region (Figure 1a). These measurements were performed at aerobic conditions with oxygen from the ambient atmosphere dissolved in the reaction mixture, and at anaerobic conditions when oxygen was removed by argon gas. For the later experiments oxygen was removed from the standard solutions by purging with argon gas for 20 min, after which the solutions were tightly sealed for later use. Cuvettes were also filled with argon and then sealed. To ensure oxygen free reactions cuvette filling was performed under a gentle argon gas stream and sealed with a cuvette cap. All measurements were performed at least 3 times. Curves represented in graphs are average values of repeated measurements.

Samples for MALDI ToF and FIB SEM analysis after the synthesis were collected by filtering through Milipore polycarbonate filters (pore size 0.22 µm) and lyophilized prior to the analysis. For MALDI ToF measurements Ppy powder (7–10 mg/mL) was dissolved in methanol, tetrahydrofuran, and dimethyl sulphone mixture at a ratio of 49:49:2. To assist solvation samples were sonicated in an ultrasound bath for 1 h at 60 °C. It was observed that only a small part of Ppy had dissolved, possibly around 5% of the total mass. The obtained Ppy suspension was diluted with the matrix compound *trans*-2-[3-(4-*tert*-Butylphenyl)-2-methyl-2-propenylidene]malononitrile (DCTB, *M_r_* = 250.6 g/mol) methanol based solution (30 mg/mL) [41] up to a 1:10 ratio. Samples with diluted Ppy were then transferred on a MALDI ToF plate and left to crystalize before analysis. Using the linear MALDI ToF detector Ppy was sampled in all possible mass intervals from 500 Da to 210 kDa.

For FIB SEM images Ppy samples were suspended in methanol and suspension transferred on microscopic glass. Samples were coated with a thin chrome layer prior to visualization.

## 3. Results and Discussion

Dissolved [Fe(CN)_6_]^3−^ acted as an oxidant which induced the polymerization of pyrrole in both oxygen-containing and oxygen-free solutions. In the samples containing pyrrole and [Fe(CN)_6_]^4−^ the formation of Ppy was observed only when oxygen was present in the solution. All the related experiments and kinetics of the polymerization of pyrrole are evaluated and described below.

### 3.1. Polypyrrole Formation

In the first part of this research the formation of Ppy was evaluated under different combinations of initial concentrations of compounds (Table 1). As expected [Fe(CN)_6_]^3−^ acting as an oxidant resulted in much faster formation of Ppy (Table 1, sample No. 1) in comparison with the formation rate observed in [Fe(CN)_6_]^4−^ solution (Table 1, sample No. 2). Ppy formation was much slower in the presence of [Fe(CN)_6_]^4−^ because [Fe(CN)_6_]^4−^ first needs to be oxidized into [Fe(CN)_6_]^3−^ by dissolved oxygen. It is assumed that [Fe(CN)_6_]^4−^ cannot initiate the polymerization of pyrrole before it is oxidized (Table 1, sample No. 2). To prove this assumption spectrophotometric evaluation of Ppy formation was conducted.

### 3.2. Spectrophotometric Evaluation of Ppy Formation Initiated by [Fe(CN)_6_]^3−^

[Fe(CN)_6_]^3−^ initiated Ppy formation was evaluated by UV-Vis spectrophotometry. Polymerization experiments were started by adding pyrrole into a reaction buffer containing [Fe(CN)_6_]^3−^ or [Fe(CN)_6_]^4−^.

Measurements were performed at wavelength ranges where selected compounds showed almost independent optical absorption. Spectra of [Fe(CN)_6_]^3−^, [Fe(CN)_6_]^4−^, and Ppy, which was formed using [Fe(CN)_6_]^3−^, were measured in the range between 300 and 900 nm (Figure 1). [Fe(CN)_6_]^3−^ has an absorption maximum at 420 nm (Figure 1a). [Fe(CN)_6_]^4−^ does not show any distinct absorption maximum in the visible light region (Figure 1a). A poorly expressed optical absorption maximum of Ppy was recorded at 460 nm (Figure 1b) which is in line with data reported in other researches [9,42]. As is described in the literature this is a typical double bond absorption, which is measurable when delocalized electrons are transferred from bonding orbitals to non-bonding orbitals (π-π*), though this type of absorption is only characteristic of relatively short chains or small particles of Ppy [42]. Ppy spectra were recorded in different samples with varying concentrations of initial materials to evaluate if the absorption maximum of Ppy remained at a constant wavelength. Results show that the absorption maximum is constant and does not shift sideways, although some unspecific absorbance was observed. We presume that this absorption in a wide range of wavelengths appeared due to light scattering combined with other secondary effects induced by larger Ppy particles. In all these reactions no additional chemicals which can affect Ppy particle size were used, however due to a relatively high oxidizer ([Fe(CN)_6_]^3−^) concentration the formation of Ppy particles of different size was observed.

**Figure 2:**
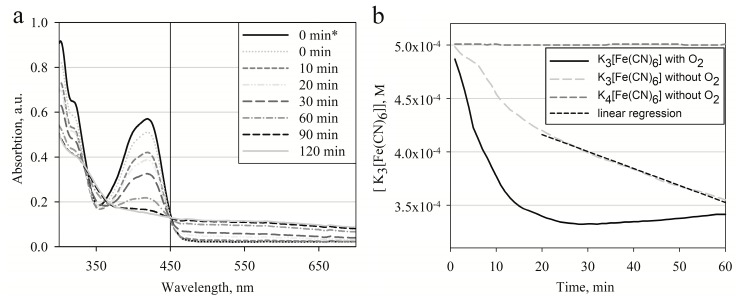
(**a**) Change of absorption spectra of a sample containing 0.5 mM [Fe(CN)_6_]^3−^ and 0.5 M of pyrrole at different incubation periods; (**b**) evolution of absorption at 420 nm vs. incubation time in a solution consisting of 0.5 M of pyrrole with either 0.5 mM of [Fe(CN)_6_]^3−^ or 0.5 mM of [Fe(CN)_6_]^4−^ at pH 7.0, under aerobic and anaerobic conditions. Linear regression from t = 20 min was observed with *R*^2^ = 0.9933; 0 min*, spectra registered before the addition of Py into reaction mixture.

Further changes of spectra during the course of polymerization initiated by [Fe(CN)_6_]^3−^ were inspected. Polymerization was performed in 0.1 M PBS, pH 7.0, with 0.5 mM of [Fe(CN)_6_]^3−^ and 0.5 M of pyrrole. Absorption spectra were registered at chosen time intervals (Figure 2a). In this experiment optical absorption at λ = 420 nm has decreased indicating that [Fe(CN)_6_]^3−^ is involved in the reaction with pyrrole monomers and is reduced by pyrrole. The origin of optical absorbance increases in the range from 450 to 700 nm which is most probably related to the interconnection of shorter Ppy polymer chains. Such prolongation of conjugated double bond systems induces a bathochromic absorption shift. In this particular experiment the polymerization reaction initiated with 0.5 mM [Fe(CN)_6_]^3−^ was relatively slow. In Figure 2a an isosbestic point at λ = 450 nm was observed. This indicates that during a time frame lasting up to 2 h from initialization of the polymerization reaction the spectra of [Fe(CN)_6_]^3−^ and Ppy are still not overlaying one another. This allows spectrophotometric measurement of both [Fe(CN)_6_]^3−^ and Ppy independently.

Similar experiments were performed measuring optical absorption at λ = 420 nm while the reaction was performed at aerobic and anaerobic conditions. In these experiments the reduction of [Fe(CN)_6_]^3−^ by pyrrole to [Fe(CN)_6_]^4−^ was observed. In Figure 2b curves which indicate [Fe(CN)_6_]^3−^ concentration changing in time at aerobic and anaerobic conditions are presented. In a reaction mixture which contained 0.5 M of pyrrole and 0.5 mM of [Fe(CN)_6_]^3−^ at aerobic conditions the process reaches equilibrium during the 35th minute after the initialization of the polymerization reaction. The formed [Fe(CN)_6_]^4−^ is re-oxidized back into [Fe(CN)_6_]^3−^ by oxygen dissolved in the solution and the concentration of [Fe(CN)_6_]^3−^ remains constant after this point. The concentration of dissolved oxygen in a reaction mixture of 0.1 M PBS, pH 7.0, at 25 °C is ~250 µM [43,44]. Alternatively under anaerobic conditions [Fe(CN)_6_]^3−^ reacts slowly with pyrrole and the concentration of [Fe(CN)_6_]^3−^ steadily decreases. The decrease of [Fe(CN)_6_]^3−^ concentration from the 20th minute after the initialization of polymerization reaction can be approximated using a linear function with regression coefficient *R*^2^ > 0.99. This suggests that during polymerization the formed [Fe(CN)_6_]^4−^ is not oxidized back into [Fe(CN)_6_]^3−^. From this graph it is possible to determine that at anaerobic conditions with an excess of pyrrole the polymerization reaction is terminated when all [Fe(CN)_6_]^3−^ is consumed or has reached its lowest thermodynamically favorable concentration. At aerobic conditions the cycling of oxidation and reduction reactions take place, which only stop when all pyrrole has been polymerized into polypyrrole. In both reactions Ppy formation was observed, however this is not represented in figures. In anaerobic conditions the reaction between [Fe(CN)_6_]^4−^ and pyrrole was also inspected and no transition or oxidation from [Fe(CN)_6_]^4−^ to [Fe(CN)_6_]^3−^ or formation of Ppy was observed (Figure 2b). Constant oxidation of [Fe(CN)_6_]^4−^ into [Fe(CN)_6_]^3−^ at aerobic conditions without Py was also observed, although the data is not presented. The experiment described here proves that the re-oxidation step of [Fe(CN)_6_]^4−^ is essential in order to produce Ppy efficiently at relatively low initial concentrations of [Fe(CN)_6_]^3−^. This shows that the [Fe(CN)_6_]^3−^/[Fe(CN)_6_]^4−^ is reusable in the initiation of Ppy polymerization reactions. Few studies have pointed out that iron compounds such as FeCl_3_ and Fe_2_O_3_ can be regenerated and reused in Ppy and polyaniline synthesis when they are coupled with an oxidation cycle by oxygen [45,46].

**Figure 3:**
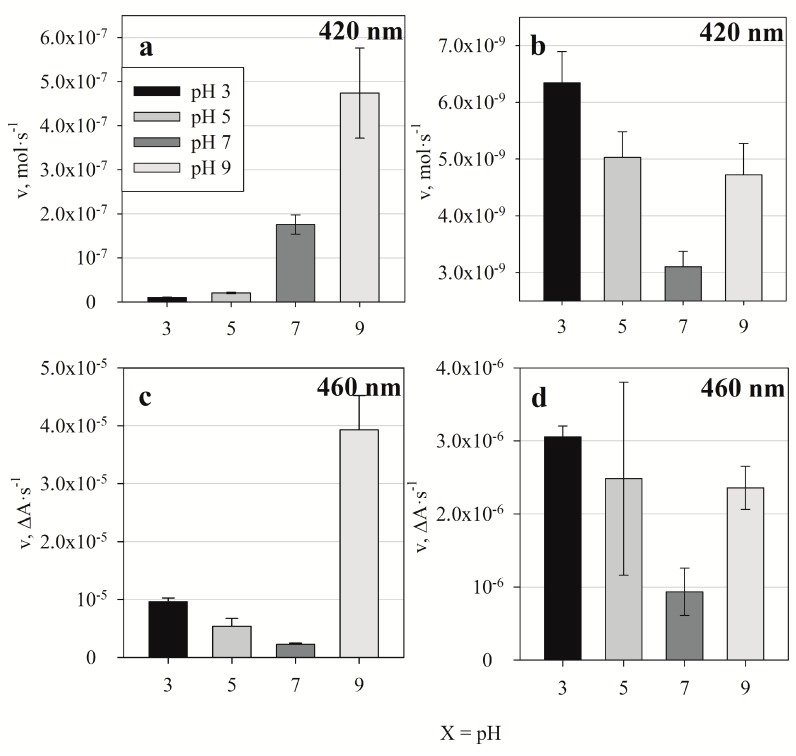
Reaction rates at different pH values: (**a**) [Fe(CN)_6_]^3−^ concentration decrease rate was measured at 420 nm in the presence of [Fe(CN)_6_]^3−^ and pyrrole due to the formation of Ppy; (**b**) [Fe(CN)_6_]^3−^ concentration increase rate due to the re-oxidation of [Fe(CN)_6_]^4−^ by dissolved oxygen was measured at λ = 420 nm; (**c**) Ppy polymerization rate in the presence of [Fe(CN)_6_]^3−^ measured at λ = 460 nm; (**d**) Ppy polymerization rate in the presence of [Fe(CN)_6_]^4−^ at 460 nm. In all samples 0.5 M of pyrrole and either 0.5 mM of [Fe(CN)_6_]^3−^ or 0.5 mM of [Fe(CN)_6_]^4−^ was used. All reactions were performed in aerobic conditions. Error bars indicates standard deviation of the calculated reaction rate.

As the above shows, the re-oxidation of [Fe(CN)_6_]^4−^ into [Fe(CN)_6_]^3−^ has been performed by oxygen dissolved in PBS under standard conditions. To control this process it is important to find conditions where the influence of dissolved oxygen on the reaction is the lowest or has no impact at all. In Figure 3 graphs represent both the variation rate of [Fe(CN)_6_]^3−^ concentration and the rate of Ppy formation at different pH values measured at 420 and 460 nm. Measurements were performed in a 0.1 M PBS of different pHs. 0.5 M of pyrrole was present in all these reaction mixtures and 0.5 mM of [Fe(CN)_6_]^3−^ or 0.5 mM of [Fe(CN)_6_]^4−^ were added into the polymerization solution. At different pH values we observed polymerization of Ppy using [Fe(CN)_6_]^3−^ according to reaction Equation (1).

Py + [Fe(CN)_6_]^3−^ → Ppy + [Fe(CN)_6_]^4−^ (1)

In this polymerization reaction (Equation (1)), the rate of [Fe(CN)_6_]^3−^ concentration change through time was evaluated and is represented in Figure 3a. The Ppy formation rate was evaluated and is represented in Figure 3c. The other two graphs represent kinetic parameters of the subsequent cascade of reactions (Equation (2)):

(2) $${\left[\mathrm{Fe}{\left(\mathrm{CN}\right)}_{6}\right]}^{4-}\overset{O_{2}}{\to }\;{\left[\mathrm{Fe}{\left(\mathrm{CN}\right)}_{6}\right]}^{3-}\;\overset{\mathrm{Py}}{\to }\;\mathrm{Ppy}+{\left[\mathrm{Fe}{\left(\mathrm{CN}\right)}_{6}\right]}^{4-}$$

Figure 3b represents the rate of change of [Fe(CN)_6_]^3−^ concentration and Figure 3d addresses the formation rate of Ppy. In Figure 3b the represented rates correspond to changes of overall [Fe(CN)_6_]^3−^ concentration. During the experiment a constant increase of [Fe(CN)_6_]^3−^ concentration was observed, illustrating that the oxidation of [Fe(CN)_6_]^4−^ to [Fe(CN)_6_]^3−^ is faster than the reduction of [Fe(CN)_6_]^3−^ by Ppy. In Figure 3a, the rates of [Fe(CN)_6_]^3−^ concentration decrease (*v* = 1.75 × 10^−7^ mol·s^−1^ and *v* = 4.74 × 10^−7^ mol·s^−1^) indicate that the reaction is faster at neutral pH (pH 7.0) and at basic conditions (pH 9.0). In Figure 3b the formation of [Fe(CN)_6_]^3−^ from [Fe(CN)_6_]^4−^ due to oxidation by dissolved ambient oxygen is represented. The slowest oxidation of [Fe(CN)_6_]^4−^ into [Fe(CN)_6_]^3−^ occurs at pH 7.0 with a reaction rate of *v* = 3.10 × 10^−9^ mol·s^−1^. This process is approximately two orders of magnitude slower than that described above. Figure 3c,d represent Ppy formation rates in the presence of [Fe(CN)_6_]^3−^ and [Fe(CN)_6_]^4−^ respectively. In both cases polypyrrole particles are formed the slowest at pH 7.0. In the presence 0.5 mM of [Fe(CN)_6_]^3−^ the reaction rate was *v* = 2.24 × 10^−6^ ∆A·s^−1^, and in the presence 0.5 mM of [Fe(CN)_6_]^4−^ it was equal to *v* = 9.35 × 10^−7^ ∆A·s^−1^. From these results it can be estimated that if [Fe(CN)_6_]^4−^ is in the same reaction mixture with Py at aerobic conditions and at pH 7.0 then the oxidation process (Equation (2)) is the rate determining step for the whole polymerization process. Consequently, the variation of pH can be exploited to control reaction kinetics.

Differences in Ppy formation rates determined at different pH values suggest that the polymer might undergo polymerization under two different mechanisms. According to the detailed Ppy synthesis mechanism proposed in [15] there are few steps during the prolongation of Ppy chains when formed Ppy undergoes deprotonation. In the basic pH range hydroxyl ions might promote this deprotonation process. However, in an acidic medium deprotonation is slower, therefore the velocity of Ppy prolongation is also slower than the initiation of Ppy chain formation where protonation is important, therefore short Ppy oligomers are formed more rapidly. This could be the reason why reaction rates were almost 4 times higher in basic solutions than those in acidic solutions (Figure 3c). In the case of Ppy chain prolongation longer Ppy chains absorb visible light mostly at 460 nm. The increased acidity facilitates the polymerization initiation step during which short Ppy oligomer chains appear more rapidly than the long ones.

### 3.3. Ppy Doping in Acidic Medium and Characterization of Aggregates

**Figure 4:**
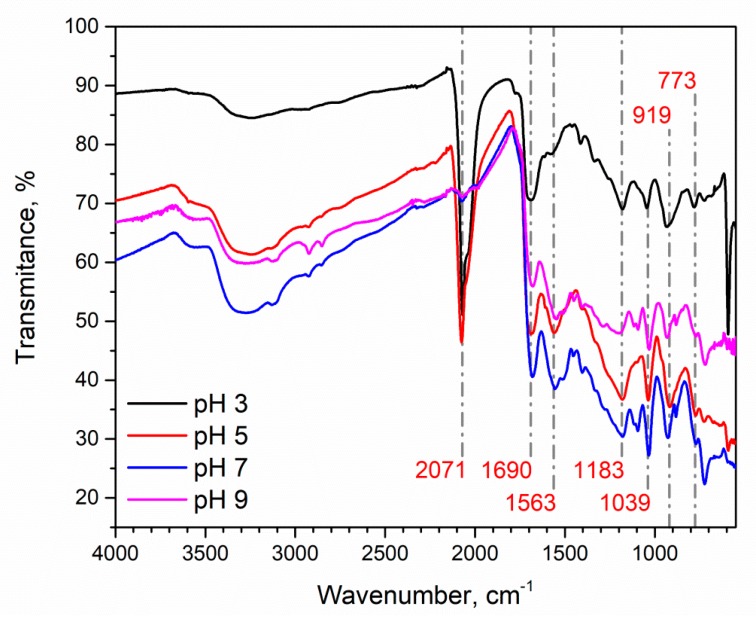
FTIR spectra of Ppy synthesized at different pH values. 0.5 M of Py and 0.04 M of K_3_[Fe(CN)_6_] were used for Ppy synthesis in 0.1 M PBS buffers of four different pH’s: 3.0, 5.0, 7.0 and 9.0.

The polypyrrole powders prepared at different pH values were analysed by FTIR. All Ppy samples were synthesized using K_3_[Fe(CN)_6_] in PBS at pH values of 3.0, 5.0, 7.0 and 9.0. FTIR spectra (Figure 4) showed main characteristics peaks at 774, 1039, 1183, 1563, and 1690 cm^−1^ and a broad band at ~3100–3500 cm^−1^. These peaks were observed in all samples. A variable peak at 2071 cm^−1^ has been observed in Ppy which was formed in an acidic polymerization solution. The main FTIR characteristic peaks are in good agreement with that presented by other researchers [38]. The bands at 774 cm^−1^ and 1684 cm^−1^, which are ascribed to C-N bond, and 1563 cm^−1^ correspond to fundamental vibrations of the polypyrrole ring. The band at 1039 cm^−^^1^ is based on the =C–H in-plane vibrations, and the band at 1196 cm^−^^1^ corresponds to the C–N stretching vibrations. The peak at ~3100–3500 cm^−1^ corresponds to the N–H bond. The band at 2071 cm^−1^ corresponds to C-N stretching of K_x_[Fe(CN)_6_]. The absorption band for [Fe(CN)_6_]^x−^ varies between 2040–2120 cm^−1^ [47]. It is most likely that this band represents [Fe(CN)_6_]^4−^ [48]. These FTIR spectra suggests that [Fe(CN)_6_]^4−^ remains in the Ppy layer as a dopant if Ppy is synthesized in an acidic medium. This is a common feature for doping of Ppy in an acidic medium because Ppy becomes positively charged and can incorporate negative ions in its structure [47]. Consequently, the efficiency of Ppy doping by [Fe(CN)_6_]^4−^ can be controlled by the varying the pH of the polymerization solution.

Analysis of FTIR spectra reveals that it is possible to achieve doping with [Fe(CN)_6_]^4−^ directly without using an additional oxidizer or an external electric stimulus, and to produce the Ppy/[Fe(CN)_6_]^4−^ composite in one step, which corresponds to results published in [49], where additional oxidizer was used. This finding corresponds to that presented in other research, which reports that in acidic mediums anions tends to remain within the Ppy matrix [50].

**Figure 5:**
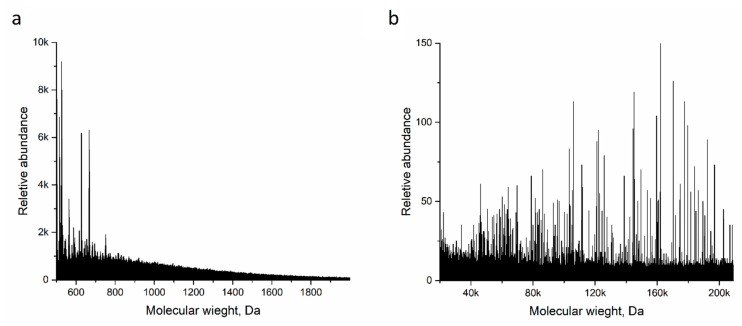
MALDI ToF spectra in different mass ranges. Spectra were registered in a DCTB matrix using a linear detector. Only those mass ranges in which some particle abundance was observed are represented here. (**a**) mass range of 500–2000 Da, (**b**) mass range of 20k–210k Da.

Synthesized Ppy was also inspected with a focused ion beam scanning electron microscope (FIB SEM) and matrix-assisted laser desorption/ionization time of flight (MALDI ToF). It was determined that Ppy, which was prepared in basic or neutral conditions, is only partially soluble in organic solvents. This is most probably due to Ppy having a neutral nature at this pH, meaning that the Ppy polymeric-backbone was not in an ionic state and thus was partially soluble. From MALDI ToF results (Figure 5a) it was determined that particles with molecular mass between 550 to 1000 Da are highly abundant. This suggests that only Ppy oligomers tend to dissolve and form co-crystals with *trans*-2-[3-(4-*tert*-Butylphenyl)-2-methyl-2-propenylidene]malononitrile (DCTB). High molecular mass Ppy was also observed with molecular mass around 160 kDa (Figure 5b). According to [51] polymers such as Ppy cannot have their molecular mass determined using MALDI ToF due to the fact that these type of polymers are insoluble in any type of solvents. However, according to [41] it is possible to measure polythiophenes, similar class to Ppy polymers, using DCTB as a supporting matrix compound. Using DTCB as a matrix we were only able to determine with confidence Ppy with low molecular mass. According to our best knowledge, no other studies provided any convincing evidence where Ppy molecular mass was determined using MALDI ToF.

**Figure 6:**
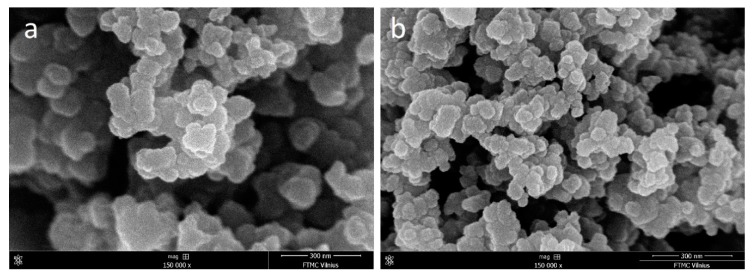
FIB SEM images of Ppy aggregates synthesized using 0.5 M Py and 0.04 M K_3_[Fe(CN)_6_]. (**a**) Synthesis was performed in 0.1 M PBS, pH 3.0; (**b**) synthesis was performed in 0.1 M PBS, pH 9.0. Both images were obtained using 150k magnification.

FIB SEM images revealed that commonly reported Ppy aggregates constituted from smaller spherical shaped units (Figure 6). Upon further inspection variation among spherical unit size was detected, which was altered by pH value. Ppy prepared at different pHs (3.0, 5.0, 7.0 and 9.0) were formed respectively of 152 ± 12 nm, 76 ± 26 nm, 52 ± 15 nm and 54 ± 14 nm size base units. Thus, Ppy synthesized in more acidic conditions tended to form larger Ppy units. On the other hand, the Ppy unit size was lower at neutral and basic conditions. FIB SEM study in agreement with FTIR data suggests that the incorporation of [Fe(CN)_6_]^4−^ into the Ppy structure increases its porosity. This is possible because formed Ppy(H^+^)/[Fe(CN)_6_]^4−^ ion pairs in acidic medium, in accordance with FTIR results, might be hydrated and yield larger constituent units of Ppy.

## 4. Conclusions

In this research we report the evaluation of polymerization of pyrrole initiated by [Fe(CN)_6_]^3−^. It was determined that if [Fe(CN)_6_]^4−^ is initially added into a bulk polymerization solution then it can be oxidized into [Fe(CN)_6_]^3−^ by dissolved oxygen. The formed [Fe(CN)_6_]^3−^ then acts as the initiator of the pyrrole polymerization reaction. On the other hand using [Fe(CN)_6_]^3−^ the polymerization rate of pyrrole can be controlled by varying the pH value of the polymerization bulk solution. There is some evidence that Ppy-based particle size increases by doping with [Fe(CN)_6_]^4−^ and such doping is more efficient in an acidic medium. The slowest Ppy formation rate was observed when the reaction was performed at neutral pH (pH 7.0).

## Figures and Tables

**Table 1:** Visual evaluation of Ppy (dark precipitant) formation in solutions with different compositions, performed at 0.04 M concentration of [Fe(CN)_6_]^3−^ or [Fe(CN)_6_]^4−^ with 0.5 M of pyrrole.

Sample No.	Compositions			Description of Result
	Fe(CN)_6_]^3−^	[Fe(CN)_6_]^4−^	Pyrrole	
1	0.04 M	-	0.5 M	Ppy was formed instantly
2	-	0.04 M	0.5 M	Ppy distinctly appeared only after 1–2 days
